# Volume XXII

**Published:** 1882-08

**Authors:** 


### Editorial.

---

### Volume XXII.

With the present number the Journal enters upon its twenty-second volume. We leave our patrons and readers who have contributed to, or scanned its pages, and thus become directly interested in our labors, to render judgment upon the merits of the past. It may be becoming the editors to speak of the future; and while sharing with the profession whatever praise or censure may be given the Journal in the efforts to advance the interests of medicine, we may affirm that, as the medical press is but the reflex of the medical thought of its constituency, the future of the Journal will depend not only upon the energy and zeal of its editorial corps, but upon the profession for which it aims to be an exponent, and whose interests it strives to advance. It is a truism that a profession which is alive to the great responsibilities devolving upon it, and earnest in its endeavors to advance the science of which it is the custodian, will seek expression for its zeal through the medium of the press. If a publication, such as this, fails to command support and to obtain a high standing, the failure then must be shared by the profession. The future of the Journal in so far as it adds to the stock of human knowledge, and to the advancement of medical science, will be largely influenced by the interested efforts of its readers and patrons.

With a view to secure such co-operation, special inducements are offered for contributions on subjects of scientific and professional interest. Under the head of Original Communications, articles from our best writers will be published, and we have assurances that this volume will surpass any of the preceding volumes in the completeness and value of its pages.

Special attention is directed to Clinical Reports, under which head the record of rare and interesting cases of post-mortem examinations, comparative results of medicinal agents, and also data gathered by the busy practitioner who finds but limited leisure for recording his professional experience, will be published.

The Translations and the Selections from our exchanges will be made solely with a view to their practical value and benefit to the profession.

Upon subjects of general professional interest, the position of the JOURNAL will be independent and its criticism will be without fear or favor. We are earnest advocates of a higher standard for medical education, believing that the Degree of Doctor of Medicine, in many of our medical colleges, is bestowed too often from considerations other than the professional attainments of the applicant.

With a larger circulation than ever before in its history, the JOURNAL finds in a wide and appreciative constituency, a field for labor and effort, which will repay for the most assiduous care, upon the part of its editors, in the preparation of its monthly issues.

We ask the active, earnest co-operation of the profession to maintain a higher standard of merit for the JOURNAL, and with such liberal assistance from our readers, the present volume will surpass any of its predecessors in professional and scientific value.

